# NOS-like activity of CeO_2_ nanozymes contributes to diminishing the vascular plaques

**DOI:** 10.1186/s12951-023-02276-5

**Published:** 2024-01-03

**Authors:** Yuxiang Sun, Tianze Xu, Yike Qian, Qiaoyun Chen, Fei Xiong, Wenxian Du, Li Xu

**Affiliations:** 1 Institute of Translational Medicine, Medical College, Yangzhou University, Yangzhou, 225001 PR China; 2 Jiangsu Key Laboratory of Integrated Traditional Chinese and Western Medicine for Prevention and Treatment of Senile Diseases, Yangzhou University, Yangzhou, PR China; 3 Department of Vascular Surgery, the Affiliated Drum Tower Hospital of Nanjing University Medical School, Nanjing, China; 4 State Key Laboratory of Bioelectronics, Jiangsu Key Laboratory for Biomaterials and Devices, School of Biological Science and Medical Engineering & Collaborative Innovation Center of Suzhou Nano-Science and Technology, Southeast University, Nanjing, 210096 People’s Republic of China; 5 Institute of Diagnostic and Interventional Radiology, Shanghai Sixth People’s Hospital, School of Medicine, Shanghai Jiaotong University, No. 600, Yishan Road, Xuhui District, Shanghai, 200233 China

## Abstract

Ceria nanoparticles (CeO_2_NPs) exhibit great potential in cardiovascular disease and nonalcoholic fatty liver disease due to its excellent antioxidant capacity. However, the profitable effect of CeO_2_NPs on many diseases is almost all attributed to the regulation of ROS. Apart from the general antioxidant function, there seems to be no more distinct mechanism to reflect its unique multi-disease improvement effect. Here, we for the first time reveal a new discovery of CeO_2_NPs in mimicking nitric oxide synthase (NOS) by catalyzing L-arginine (L-Arg) to produce nitric oxide (NO) or the derivatives. NOS-like activity of CeO_2_NPs is original and associated with multiple factors like substrate concentration, pH, temperature and time, etc. where oxygen vacancy ratio plays a more critical role. Meanwhile, NOS-like activity of CeO_2_NPs successfully elevates NO secretion in endothelial cells and macrophages without expanding eNOS/iNOS expression. Importantly, NOS-like activity of CeO_2_NPs and the responsive endogenous NO promote the re-distribution of blood lipids and stabilize eNOS expression but suppress iNOS, thus collectively alleviate the accumulation of vascular plaque. Altogether, we provide a new angle of view to survey the outstanding potential of CeO_2_NPs, apart from the inevitable antioxidant capacity, the covert but possible and more critical NOS-like enzymatic activity is more noteworthy.

**Supplementary Information:**

The online version contains supplementary material available at 10.1186/s12951-023-02276-5.

## Background

Although the formation mechanism of vascular plaques is still uncertain, excessive cholesterol and lipoprotein deposition are important driving forces that induce plaque formation [1, 2]. Accumulation of lipids on the inner wall of arteries and the formation of intimal plaques will further lead to luminal stenosis, vascular sclerosis and even atherosclerosis [3, 4]. Additionally, the secondary free radical injury and pro-inflammatory after plaque formation are the main potential mechanisms that aggravate atherosclerosis [5, 6]. As an endothelium-derived relaxing factor, nitric oxide (NO) has been shown whose physiological imbalance is another important reason for the formation of vascular plaques [5]. Endothelial NO can diffuse from endothelial cells to the underlying smooth muscle cells and induce vasodilation by stimulating the NO sensitive guanylate cyclase. NO also can diffuse into the bloodstream and inhibit platelet aggregation and adhesion [7].

However, excessive lipids and abnormal blood flow limit the NO bioavailability and disorder the function of vasodilation by weakening the shear force of blood flow and elevating oxidative stress [7, 8]. In generally, endogenous NO is synthesized by three different types of nitric oxide synthase (NOS), namely eNOS, nNOS and iNOS, while eNOS in the endothelium is activated by shear stress of the flowing blood. Although iNOS may generate large amounts of NO over long periods of time, the induced iNOS in vascular plaques is interpreted as the activation of pro-inflammatory responses [5]. Inimical iNOS will impaire NO-mediated vasodilation response by reducing NO production by eNOS or enhancing inactivation of eNOS-derived NO. Thus, supplementing the endogenous NO but restricting the adverse response of iNOS is conducive to diminishing the formation of vascular plaques by improving vasodilation ability and limiting inflammatory response.

Nanozymes have become a widely existing component in the catalytic system for various biomedical applications [9–11]. Ceria nanoparticles (CeO_2_NPs) have been widely concerned because of the mimetic enzyme activities of superoxide dismutase (SOD), catalase (CAT), oxidase and others [12, 13]. Depending on temperature and oxygen pressure, the oxidation of metal Ce and O_2_ can form many different phases including the extreme components of Ce_2_O_3_ and CeO_2_ [13]. Considering the possible electron transfer between Ce^3+^ and Ce^4+^, CeO_2_NPs are thus endowed with redox properties, oxygen storage capacity and other unknown performances. Thanks to these known and unknown properties, CeO_2_NPs have attracted widespread attention in Parkinson’s disease [14], atherosclerosis [15, 16], nonalcoholic fatty liver disease [17], etc. In the process of anti-vascular plaque, Du et al. revealed that hyaluronic acid-guided assembly of ceria nanozymes can act as the plaque-targeting ROS scavengers for anti-atherosclerotic therapy [15]. However, apart from the general antioxidant function, there seems to be no more distinct mechanism to reflect its unique multi-disease improvement effect.

**Scheme 1:**
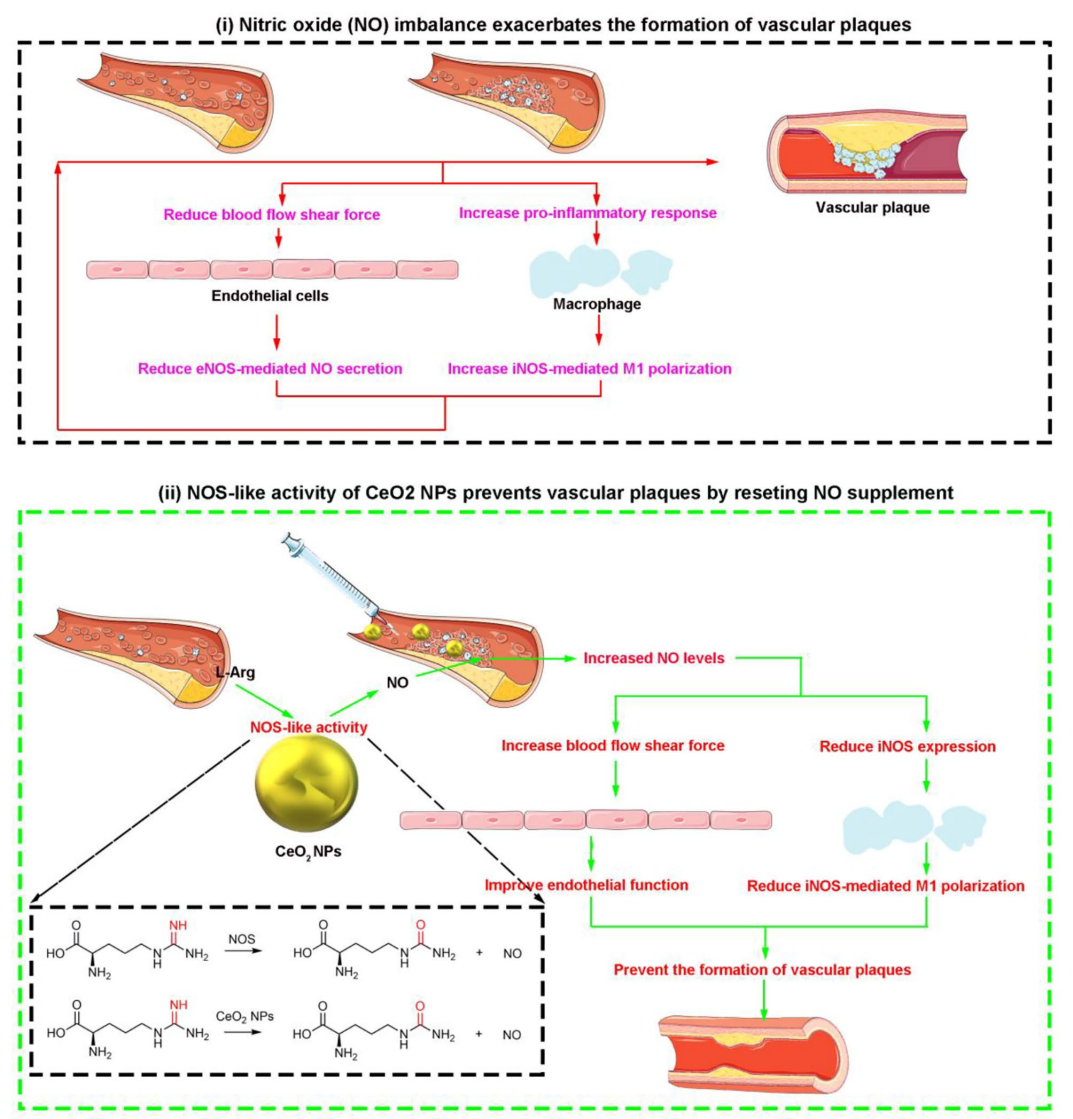
(i) illustrates that the deposition of lipids and macrophages on the vascular wall can lead to abnormal NO secretion mediated by eNOS in endothelial cells and exacerbate iNOS-mediated pro-inflammatory responses in macrophages, which in turn feedback promotes the formation of vascular plaques. (ii) illustrates that CeO_2_NPs derived NO by the NOS-like activity can prevent vascular plaque formation via improving endothelial cell function and macrophage function. CeO_2_NPs simulates the activity of NOS to elevate NO level in serum. This exogenous way to supplement endogenous NO levels continuously can change the re-distribution of blood lipids as well as cell function by altering blood flow status including blood flow shear force

In this study, we make a new discovery that dextran-guided CeO_2_NPs possess intrinsic NOS-like activity to catalyze L-arginine (L-Arg) and promote the production of NO. And the NOS-like activity of CeO_2_NPs is associated with its concentration, reaction time, pH value and temperature, although oxygen vacancy (Vo) exhibits more importance in determining the NOS-like activity. Importantly, the NOS mimic contribution can significantly increase the level of NO content in a wide range of cells, as well as IL-4, a specific Th2 cytokine that activates M2 type macrophages to suppress inflammatory factors and functions in regulating inflammatory reaction and tissue repair. What’s more, in the ApoE knockout mice (ApoE^−/−^) fed with high-fat diet, the NOS-like activity of CeO_2_NPs alleviates the formation of vascular plaque with advantage by promoting the wide distribution of free fat in myocardial tissue, and ultimately reduces the level of iNOS in plaque and the formation of foam cells (Scheme 1). The fact is that NOS-like nanozyme activity of CeO_2_NPs reveals a new mechanism for its unique behaviours in metabolic disorders as well as a huge potential for novel applications.

## Materials and methods

### Materials

Cerium nitrate hexahydrate (C105378), Dextran (D104008), L-Arginine (A137768) were purchased from Aladdin Chemical Reagent Co. Ltd (Shanghai, China). Nitric Oxide Assay Kit (S0021S), Nitric oxide fluorescence detection probe DAF-FM DA (S0025) were all purchased from Beyotime Biotechnology Co. Ltd (Shanghai, China). Anti-iNOS (ab283655, Abcam) and Anti-CD 68 (ab201340, Abcam) were purchased from Abcam. All cells were acquired from the Type Culture Collection of the Chinese Academy of Sciences (Shanghai, China). High-fat forages (ASHF4) were got from Daiz Biotechnology Co., Ltd. The pH regulation of PBS is formulated through hydrochloric acid or sodium hydroxide.

### CeO_2_NPs synthesis

The successful synthesis of CeO_2_NPs in colloid form was attained through a hydrothermal co-precipitation methodology, refer to our previous report [12]. A blend of cerium nitrate hexahydrate and dextran (in a mass proportion of 1:3) was dissolved in deionized H_2_O and thoroughly agitated until homogenous. An optimal quantity of ammonia was subsequently added to the mixture and it was heated in a precise temperature capacity of 65℃ within a water bath. The resultant colloidal solution was subject to dialysis treatment using a dedicated dialysis bag and subsequently processed through 220 nm precision filtration membranes.

### Characterization

The transmission electron microscopy (TEM) analysis was executed on a JEOL 2100 microscope. The quantification of Ce was performed with inductively coupled plasma-optical emission spectroscopy (ICP-OES), using the Perkin Elmer Optima 4300 DV, Shelton, CT instrument. The UV − vis − NIR absorption spectra were obtained using a spectrophotometer (UV-3200 S) (Mapada, China). The hydrodynamic diameter was determined using a Zetasizer Nano-ZS (Malvern Instruments). The powder XRD patterns were acquired with an AXS D8 advance (Bruker, Germany). The microplate reader was the Infinite M200 (TECAN, Switzerland) or (BioTek, USA).

### NO determination

**Fig. 1:**
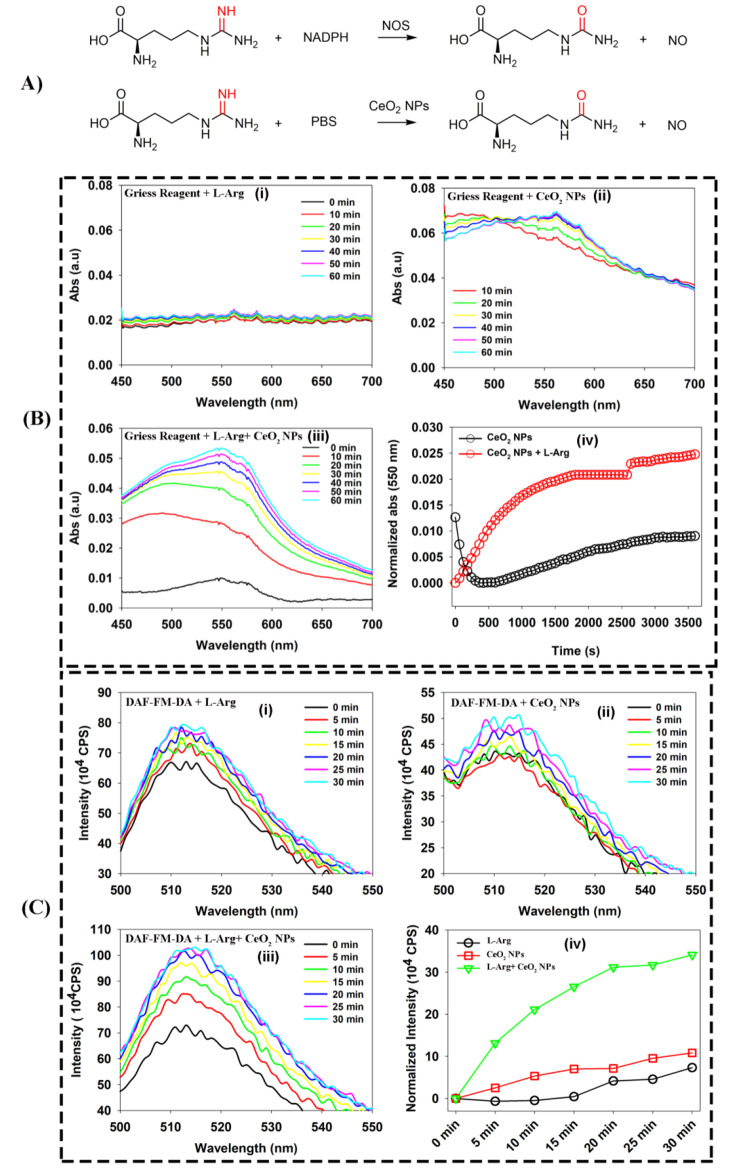
NOS-like activity of CeO_2_NPs. (**A**) Chemical equation to show nitric oxide production by CeO_2_NPs simulating NOS to catalyze L-Arg. (**B**) NO generation process is detected by Griess Reagents, all tests were performed in 0.01 M PBS buffer, where (i) is L-Arg alone, (ii) is Griess Reagents plus CeO_2_NPs, (iii) is Griess Reagents plus CeO_2_NPs and L-Arg, while (iv) shows the dynamic change process in (ii) and (iii) within 1 h. (**C**) NO generation process is detected by DAF-FM-DA, a fluorescent probe of NO, where (i) (ii) (iii) (iv) are similar to that in (**C**), but the excitation wavelength is set at 495 nm and emission wavelength of 515 nm is counted in (iv). In above tests, concentration of L-Arg is fixed at 0.29 mg/mL, the concentration of CeO_2_NPs is fixed at 1mM (Ce element)

Nitric Oxide Assay Kit (S0021S) and Nitric oxide fluorescence detection probe DAF-FM DA (S0025) were used to detect the NO level. In vitro, 900 µL Griess Reagent I and 900 µL Griess Reagent II were mixed in detection pool in advance, and 200 µL suspension with L-Arg and CeO_2_NPs was then added into the mixed Griess Reagents. As the reaction progresses, the absorbance at 630 nm was determined. For nitric oxide fluorescence detection probe DAF-FM DA, 2 mL PBS buffer, the probe concentration was fixed at 10 µM, the suspension with L-Arg and CeO_2_NPs was then added into the solution. As the reaction progresses, the fluorescence intensity of emission wavelength of 515 nm was counted by exciting at 495 nm. In Fig. 1, the concentration of L-Arg was fixed at 0.29 mg/mL, the concentration of CeO_2_NPs was fixed at 1mM (Ce element), excluding those with special markings. For the determination of NO in cells and serum, the experimental steps are carried out according to the instructions of the kits.

### Enzyme-linked immunosorbent assay (ELISA) for TNF-a and IL-4

The THP-1 and Raw264.7 cells were treated in accordance with the different experimental requirements. Following the treatment, the supernatants were collected and used to measure the levels of TNF-α (Proteintech, KE00154, KE10002) and IL-4 (Proteintech, KE00016, KE10010), as previously described. A specific instrument (BioTek, USA) was employed to measure the optical density (OD) values at a specific wavelength of 450 nm, with a standard curve drawn. Every step was carried out following the manufacturer’s specific protocols.

### Animal protocol

ApoE knock-out C57BL/6 mice (ApoE^−/−^) were provided by the Jiangsu Jicui Yaokang Biotechnology Co., Ltd (China). All animal experiments were carried out conforming to the Guideline for Animal Experimentation in agreement with the animal care committee of Yangzhou University. For the in vivo study, 12-week-old male ApoE^−/−^ mice were provided with high-fat diets for a period of 20 weeks. A sub-group of these mice received CeO_2_NPs via intraperitoneal injection at a dosage of 0.6 mg/Kg (Ce element).

Additionally, the vascular dissection was carried out with the assistance of the vascular surgery team, Affiliated Drum Tower Hospital, Medical School of Nanjing University, and the oil-red staining and immunohistochemistry were carried out by pathology team of Affiliated Drum Tower Hospital, Medical School of Nanjing University. All tests were conducted in strict accordance with the implementation guidelines.

### Western blot analysis

Cultured cells were washed twice with PBS and then lysed in RIPA lysate buffer (Beyotime, Poo13C, China). Insoluble materials from cultured cell lysates were removed by a brief centrifugation at 4℃, and the supernatants were subjected to 10% SDS-polyacrylamide gel electrophoresis and transferred to a polyvinylidene difluoride filter (PVDF) membrane by a transfer apparatus at 300 mA for 1.5 h. The membrane was then blocked with 5% nonfat milk, followed incubated with primary antibody overnight at 4℃, washing and then with secondary antibodies for 2 h at room temperature (RT) and scanned with the imaging system (Tanon, 4600SF).

### Statistical analysis

The statistical analysis was carried out by SPSS software via the Student’s t-test. All of the data in this work were expressed as the mean value with standard deviation. Statistical significance was expressed as follows: * *p* < 0.05; ** *p* < 0.01; *** *p* < 0.001.

## Results and discussion

### Preparation and characterization

**Fig. 2:**
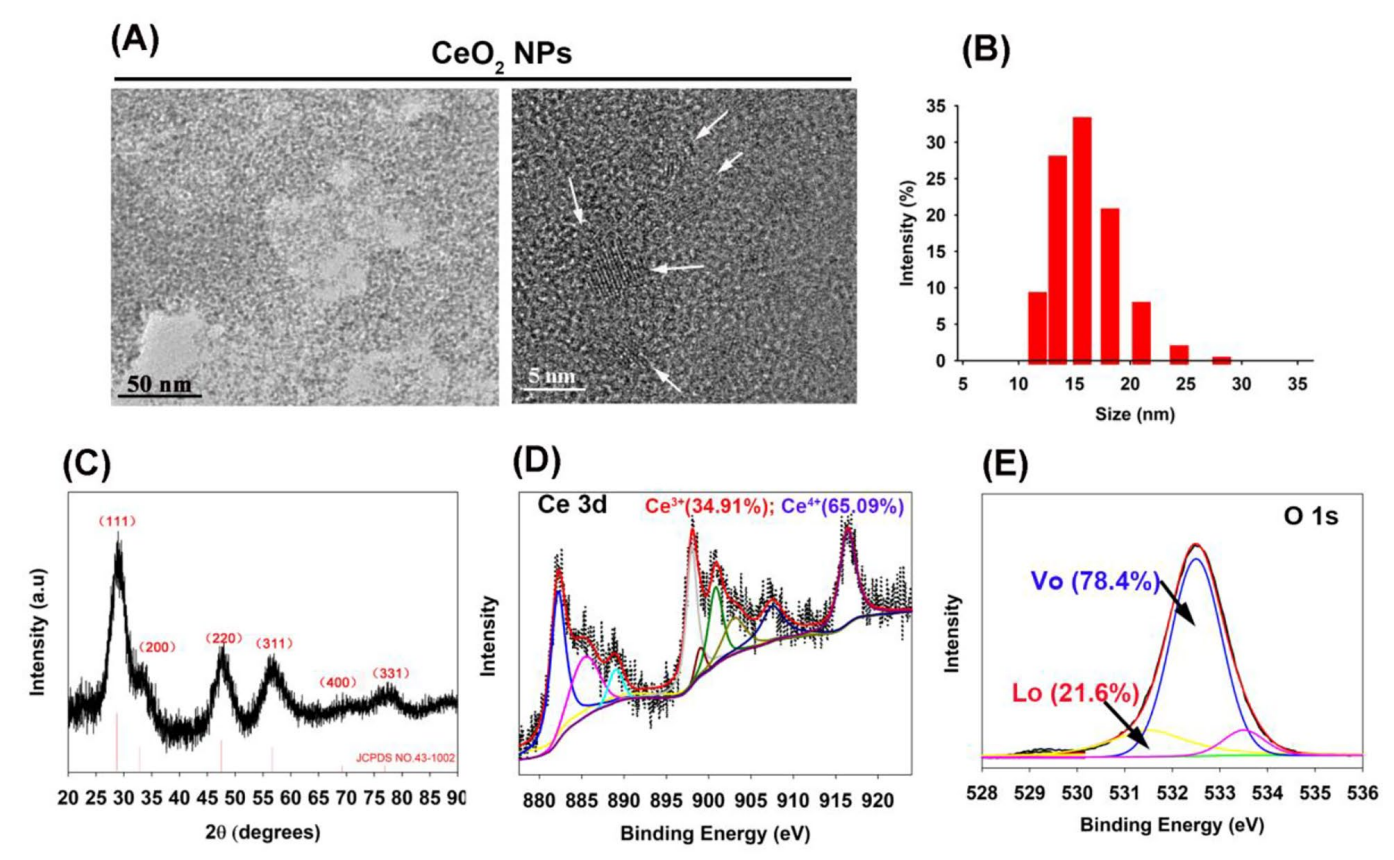
Characterization of CeO_2_NPs. (**A**) TEM of CeO_2_NPs. (**B**) Hydrodynamic size change of CeO_2_NPs aqueous solution. (**C**) X-ray diffraction (XRD) of freeze-dried powder of CeO_2_NPs. X-ray photoelectron spectroscopy (XPS) of freeze-dried powder of CeO_2_NPs and the spectrum of Ce element (**D**) and O element (**E**) are divided into peaks. The proportions of Ce^3+^ and Ce^4+^ elements and the proportions of lattice oxygen (Lo) and vacancy oxygen (Vo) of O elements are calculated after peak splitting

The preparation procedures of CeO_2_NPs is shown in previous reports and guided with dextran (Mw: 40,000) [12]. Trans-mission electron microscopy (TEM) first shows the appearance and size of CeO_2_NPs (Fig. 2A). Dynamic light scattering reveals that the hydrodynamic diameter of CeO_2_NPs is about 15 nm (Fig. 2B). After lyophilized CeO_2_NPs, the XRD pattern presents several diffraction peaks assigned to the CeO_2_NPs phase (Fig. 2C). Meanwhile, XPS data identifies the Ce element (specific binding energy of 870–925 eV) and O element (specific binding energy of 528–536 eV), where the valence state distribution of Ce includes Ce^3+^ and Ce^4+^ after peak-splitting processing (Fig. 2D), while O element exhibits the lattice oxygen (Lo) and vacancy oxygen (Vo) (Fig. 2E).

### NOS-like activity of CeO_2_NPs

Nitric oxide synthase catalyzes L-Arg to produce NO with the help of electronic donor NADPH (Fig. 1A). To demonstrate that CeO_2_NPs can catalyze L-Arg to produce NO, Griess Reagents are used to detect the generation of NO [18]. NO is unstable and tends to quickly convert to nitrite, a substance that will promote the absorbance of Griess Reagents at a specific wavelength of 550 nm. Because no nitrous acid group is introduced into the reaction system, the detected absorbance can indicate the formation of NO. In Fig. 1B, L-Arg alone shows no significant effect on the absorbance of the Griess Reagents at wavelengths between 450 and 700 nm (Fig. 1B-i). Nevertheless, CeO_2_NPs alone seems to trigger the absorption of the reaction system at 550 nm, but this change is small and unsustainable (Fig. 1B-ii). Intriguingly, when CeO_2_NPs and L-Arg co-exist within the reaction apparatus simultaneously, an augmentation in the absorbance of Griess Reagents at 550 nanometers is consistently observed (Fig. 1B-iii). Simultaneously, we further examine the dynamic progression of absorbency alteration led solely by CeO_2_NPs and subsequent reaction with (CeO_2_NPs + L-Arg), demonstrating that CeO_2_NPs facilitates continual production of NO through its catalytic influence on L-Arg (Fig. 1B-iv). .

To substantiate our aforementioned revelation, we opted for the specialized luminescent probe DAF-FM-DA to reaffirm our experimental findings [18]. The fluorescence of DAF-FM itself is only very weak, but after reacting with NO, it can exhibit strong fluorescence with an excitation wavelength of 495 nm and an emission wavelength of 515 nm. Replicating the findings by Griess Reagents, L-Arg or CeO_2_NPs alone exhibited no effect on the fluorescence emission of DAF-FM-DA when exposed to 495 nm excitation (Fig. 1C-i, ii). However, when CeO_2_NPs and L-Arg co-exist in the buffer, the system can continuously respond to excitation at 495 nm by causing an increase in the emission at 515 nm (Fig. 1C-iii). As it should be, the NOS-like activity of CeO_2_NPs derives a continuous generation of NO after interacting with L-Arg (Fig. 1C-iv).

### NOS-like activity of CeO_2_NPs is original and associated with multiple factors

**Fig. 3:**
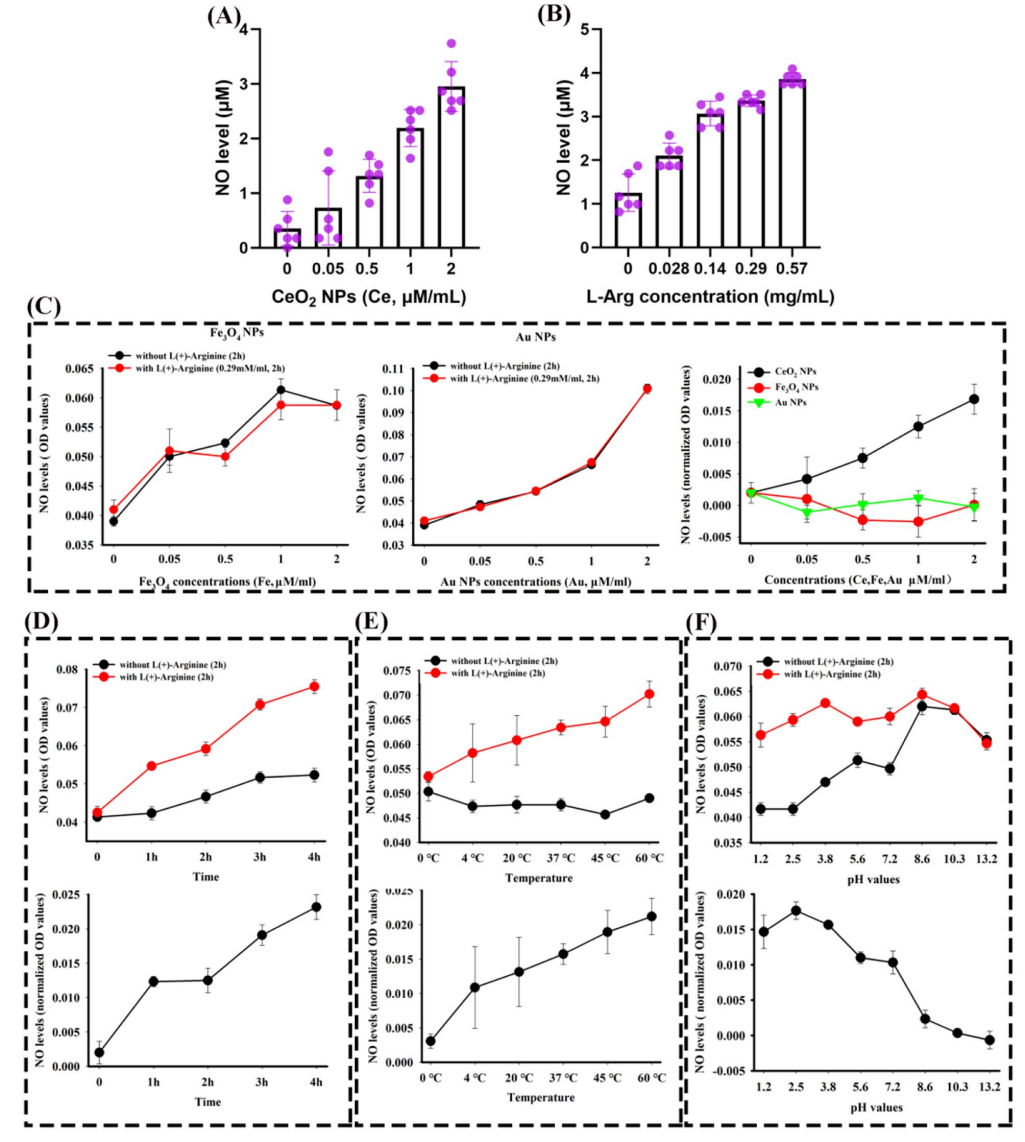
NOS-like activity of CeO_2_NPs is original and associated with multiple factors. All detection is based on Griess Reagents by counting the absorbance at 560 nm. (**A**) The concentration of L-Arg is fixed at 0.29 mg/mL, and then NO level is measured in CeO_2_NPs/L-Arg system with different CeO_2_NPs concentrations (n = 6). (**B**) The concentration of CeO_2_NPs is fixed at 1 mM (Ce element), and NO level is measured in CeO_2_NPs/L-Arg system with different L-Arg concentrations (n = 6). (**C**) As the conditions in (A), where CeO_2_NPs is replaced by Fe_3_O_4_NPs and AuNPs. L-Arg concentration is fixed at 0.29 mg/mL, CeO_2_NPs concentration is fixed at 1 mM, evaluating the effects of reaction time (**D**), temperature (**E**) and pH (**F**) on NO level (n = 3)

Subsequently, we investigate the NOS-like reactivity of CeO_2_NPs depending on Griess Reagent quantification. In the conditions where the concentration of L-Arg remains static, an increased concentration of CeO_2_NPs is observed to exert a greater influence on enhancing NO generation (Fig. S1 and Fig. 3A). Similarly, when the CeO_2_NPs concentration remains constant, an augmentation in the production of NO can also be observed alongside a rise in the L-Arg concentration (Fig. S1 and Fig. 3B). Meanwhile, we invest efforts in ascertaining similar outcomes in two additional types of nanoparticles Fe_3_O_4_NPs (Fig. S2) and AuNPs (Fig. S3), both previously demonstrated with multiple enzymatic activity [12, 19]. Regrettably, it is observed that iron (III) oxide nanoparticles and gold nanoparticles are unable to replicate the identical manifestation observed with CeO_2_NPs (Fig. 3C). Moreover, a comprehensive analysis of factors influencing the nitric oxide synthase-mimicking activity of cerium dioxide nanoparticles is conducted. Results depicted in Fig. 3D-F reveal that besides the demonstrated concentration, variables such as exposure duration, ambient temperature, pH level significantly impact the NOS-like activity of CeO_2_NPs during the generation of nitrogen oxides or their derivatives.

### Oxygen vacancy of CeO_2_NPs contributes to the NOS-like activity

The nanozyme-like activity of CeO_2_NPs is significantly influenced by the ability to switch between the Ce^3+^ and Ce^4+^ oxidation states, as well as the ability to generate and eliminate Vo [13]. The switching between Ce^3+^/Ce^4+^ corresponds to the formation and annihilation of Vo in the lattice. Interestingly, Vo of CeO_2_NPs has been demonstrated as the efficient electrocatalyst with the stabilization of the crucial intermediate of *NO via inserting into vacant sites [20]. Rona recently found CeO_2_NPs can induce the generation of NO from S-nitrosoglutathione and maintain a high NO release recovery rate by retaining their crystalline structure for at least 4 weeks [21]. Importantly, the mechanism of this newly discovered NO generation capability of CeO_2_NPs is deciphered to be attributed to the oxidation of Ce^3+^ to Ce^4+^on the surface.

**Fig. 4:**
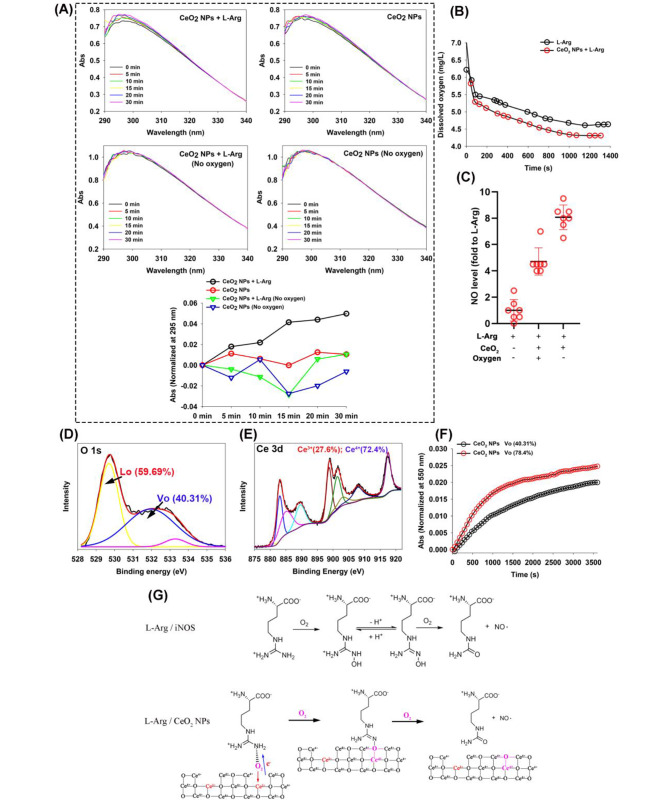
Oxygen vacancy of CeO_2_NPs contributes to the NOS-like activity. (**A**) The UV-Vis spectra of both CeO_2_NPs and CeO_2_NPs mixed with L-Arg (concentration of CeO_2_NPs is 1 mM and that of L-Arg is 0.29 mg/mL, the concentration used below is the same as here) in PBS buffer before and after nitrogen blowing are provided. (**B**) Dissolved oxygen concentration is detected by dissolved oxygen electrode, where CeO_2_NPs is added when the concentration of dissolved oxygen in L-Arg solution drops to equilibrium. (**C**) NO level is measured by Griess Reagents, and oxygen blowing is operated into PBS solution to increase dissolved oxygen level and nitrogen blowing to remove dissolved oxygen, both for 60 min (n = 7). (**D**) Another type of CeO_2_NPs is prepared according to the conditions detailed in Materials section. Post-synthesis, the synthesized CeO_2_NPs is baked at 100℃ for a total of 48 h. The XPS data obtained from the dried powder of CeO_2_NPs were classified into peaks to illustrate the spectrum of the O element (**D**) and the Ce element (**E**), in addition to the proportions of Ce^3+^/Ce^4+^ and the proportions of lattice oxygen (Lo) and vacancy oxygen (Vo). (**F**) The dynamic process of NO generation induced by CeO_2_NPs with different Vo ratio. (**G**) A possible mechanism to interpret the NOS-like activity of CeO_2_NPs

In the ultraviolet spectrum, CeO_2_NPs can cause two absorption peaks at 252 and 295 nm respectively assigned to Ce^3+^ and Ce^4+^ [12, 22]. As illustrated in Fig. S4, when CeO_2_NPs come into contacting with molecular oxygen dissolved in the buffer medium, a change in the ratio of Ce^4+^/ Ce^3+^ within the CeO_2_ crystal structure occurs, causing a gradual increase in the absorbance at 295 nm wavelength. Within the first half an hour subsequent to L-Arginine administration, the scan profile presents a more meticulous visualization of this event (Fig. 4A), suggesting that L-Arg accelerates the generation of Ce^4+^ within the lattic. It is noteworthy that the switching of Ce^4+^ caused by oxidation will not only cause the increase of absorbance at 295 nm, but also triggers the red-shift of absorbance curve between 290 and 400 nm (Fig. S5) [23]. However, in Fig. S4 and Fig. 4A, we fail to observe any significant red-shift in the absorbance curve, indicating that the process by which CeO_2_NPs catalyzes the conversion of L-Arg to NO does not resemble that of Ce^3+^ being oxidized by H_2_O_2_.That means, the rise of Ce^4+^ content in CeO_2_NPs lattice corresponding to the absorbrance at 295 nm wavelength is probably caused by the closure of oxygen vacancy (Fig. 4G).

Consistent with this results, the newly dissolved L-Arg will consume the dissolved oxygen, after the dissolved oxygen reached the equilibrium, the addition of CeO_2_NPs proceeds to consuming more dissolved oxygen in the system (Fig. 4B), indicating that the existed Vo in CeO_2_NPs competed L-Arg/O_2_ to further consume the saturated dissolved oxygen (Fig. 4B). Thus, the change in NO content was detected by simulating the lack of oxygen through nitrogen blowing in PBS buffer. The results in Fig. 4C show that the nitrogen blowing, which created an oxygen-free environment, significantly enhances the NOS-like activity to generate more NO, demonstrating a possibility that oxygen-free buffer can safeguard the Vo of CeO_2_NPs to exert the NOS-like activity. To further prove this hypothesis, we synthesize another kind of CeO_2_NPs, XPS data indicates 40.31% vacancy oxygen and 72.4% Ce^4+^, less than that of 65.09% vacancy oxygen in CeO_2_NPs synthesized previously (Fig. 4D, E). Inetrestingly, the production of NO induced by CeO_2_NPs with 40.31% Vo is significantly weaker than that induced by CeO_2_NPs with 65.09% Vo (Fig. 4F). Based on these results, we propose a mechanism as shown in the Fig. 4G to elucidate the NOS-like activity of CeO_2_NPs.

### CeO_2_NPs promote NO generation in various cells by NOS-like activity

**Fig. 5:**
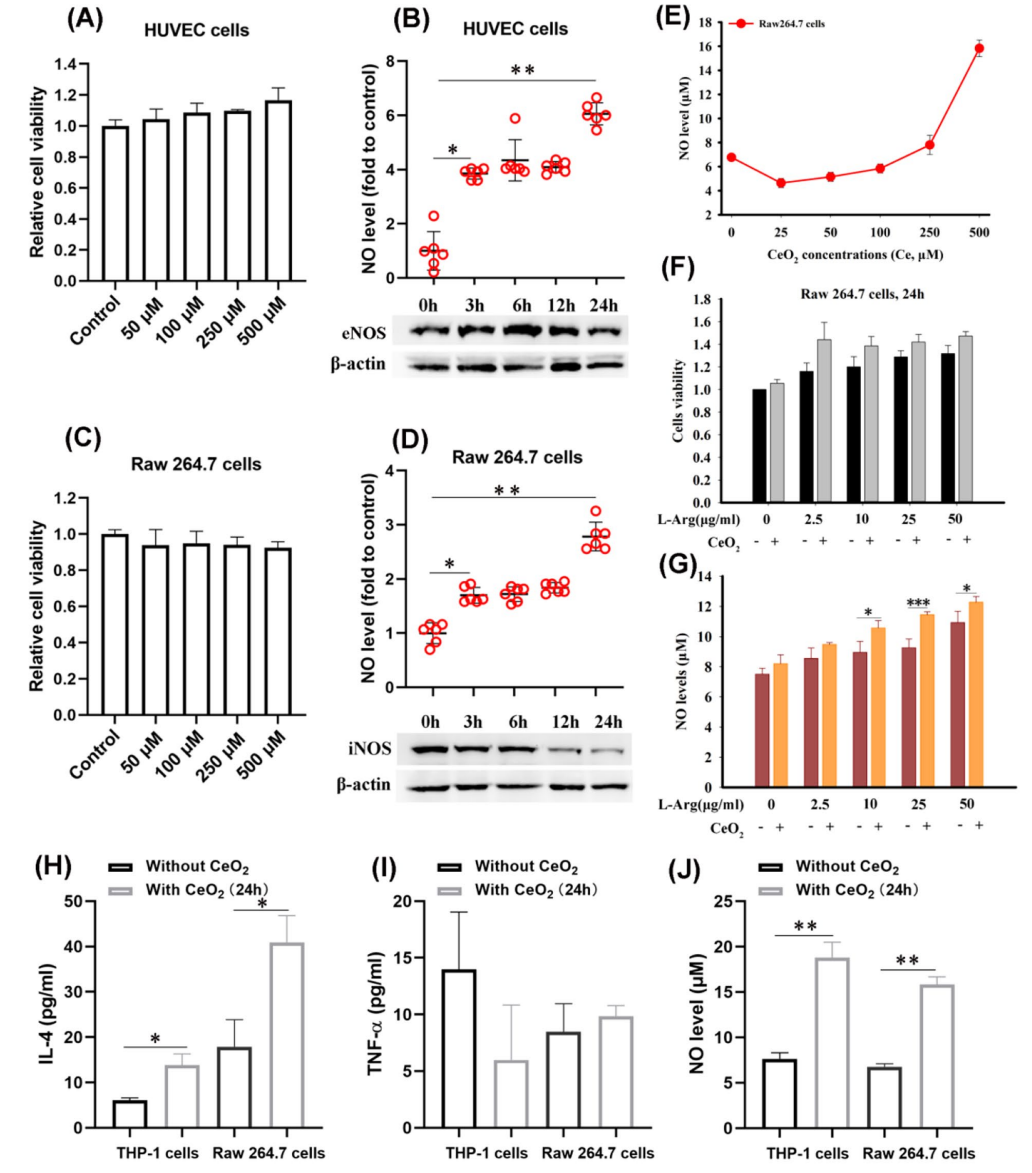
CeO_2_NPs promote NO generation in various cells by NOS-like activity. Cultured HUVEC cells and Raw 264.7 cells are exposed to varying concentrations of CeO_2_NPs for a 24 h period, with cells exposed to saline serving as a control group. Cell viability is assessed via the CCK8 assay (n = 6), as shown in (**A**) and (**C**), and the concentration of NO in the cell supernatant is measured in (**B**) and (**D**) (n = 6), along with the expression levels of the two isoforms of intracellular NO synthase (eNOS and iNOS). (**E**) In an experiment conducted on Raw 264.7 cells, the effect of treatment with varying concentrations of CeO_2_NPs for 24 h is evaluated for the presence of NO concentration (n = 3). Additionally, the survival rate of these cells post-treatment with either L-Arg at various concentrations or a combination of L-Arg and 100 µM CeO_2_NPs is determined via CCK8 assay (**F**) (n = 3), while the NO concentration in the supernatant is assessed using nitric oxide kits **(G**) (n = 3). In vitro study to evaluate the effect of 500 µM CeO_2_NPs on THP-1 and Raw 264.7 cells. The cells are exposed to CeO_2_NPs for 24 h, and the supernatant is collected subsequently. The levels of interleukin-4 (IL-4) (**H**), tumor necrosis factor-α (TNF-α) (**I**), and nitric oxide (NO) (**J**) in the supernatant are quantified by the enzyme-linked immunosorbent assay kits (ELISA) and nitric oxide kits, respectively. Data is shown as mean ± SD. *p < 0.05;**p < 0.01; ***p < 0.001

There is potential for the vasodilation of NO to improve conditions such as atherosclerosiss [5] and coronary heart disease [24]. Endothelial cells and macrophages, specifically, have been identified as the primary producers of NO in the human body [25, 26]. To investigate whether the NOS-like activity of CeO_2_NPs have a positive potential in regulating the NO generation in endothelial cells and macrophages, we examine the NO level in HUVEC cells and Raw 264.7 cells after treated by CeO_2_NPs. CeO_2_NPs of certain concentration show the good safety for these two kinds of cells (Fig. 5A, C). When HUVEC cells and Raw 264.7 cells are treated by 500 µM CeO_2_NPs (Ce element), NO secreted by cells is increased in a time-dependent manner (Fig. 5B, D). However, the intracellular expression level of eNOS and iNOS exhibits an opposite trend to that of NO, demonstrating that CeO_2_NPs can effectively control intracellular NO levels without relying solely on the alterations of NOS level. This implies that the NOS-like activity of CeO_2_NPs can contribute to intracellular NO production.

To further prove that the NOS-like activity of CeO_2_NPs is assistant to catalyze L-Arg to NO in cells, Raw 264.7 cells are co-treated by 100 µM CeO_2_NPs and different concentrations of L-Arg. 100 µM CeO_2_NPs alone shows limited impact on intracellular NO level (Fig. 5E). In comparison to the outcomes of L-Arg alone at equivalent concentration levels, after exposure to 100 µM CeO_2_NPs and varying concentrations of L-Arg in tandem, the intracellular NO level augments as the concentration of L-Arg is escalated (Fig. 5G). Additionally, the interaction of CeO_2_NPs and L-Arg also seems to promote the viability of Raw264.7 macrophage (Fig. 5F).

After phagocytosis of excessive lipid by macrophages, foam cells are formed, which will accumulate on the vascular endothelium, forming the raised plaques and then causing stenosis or even blockage of blood vessels [27]. However, the polarization of macrophages plays an important role in vascular plaque [28]. Where M1 macrophages in adipose tissue around the peripheral blood vessels of plaque are associated with a higher risk of coronary artery thrombosis, and are associated with plaque progression and unstable histological components. M2 macrophages are associated with plaque size, calcification, necrosis content and decreased number of trophoblastic tubes in the adventitia [29]. When THP-1 and Raw 264.7 macrophages are treated by 500 µM CeO_2_NPs, we observe that the secreted M2-cytokine interleukin-4 (IL-4) shows a substantial increase (Fig. 5H), whereas the M1-cytokine tumor necrosis factor-alpha (TNF-α) fails to exhibit such alteration (Fig. 5I). Inetrestingly, the increase of M2-cytokine IL-4 is highly consistent with that of NO (Fig. 5J). As shown in Fig. 5D, the elevated NO tends to inhibit the expression of iNOS after induced by the NOS-like activity of CeO_2_NPs. iNOS itself is a pro-inflammatory factor expressed in M1-type macrophage [30]. Thus, these findings suggest that CeO_2_NPs can manifest the NOS-like activity in cells and engage in regulating the phenotype of macrophages.

### CeO_2_NPs prevent vascular plaque by NOS-like activity

Given that intravascular ROS and inflammation are two characteristic features of the atherosclerotic microenvironment, CeO_2_NPs has been proposed as an appropriate strategy for atherosclerosis by synergistically regulating ROS and inflammation [31]. The relaxation of vascular smooth muscle and vasodilation induced by NO can prevent the formation of vascular plaque and reduce the accumulation of macrophages. Thus, the pharmacological stimulation of NO signal has been suggested to prevent or treat cardiovascular diseases [32]. To demonstrate that NOS-like activity of CeO_2_NPs is conducive to prevent vascular plaque, 12-week-old ApoE^−/−^ mice are fed with high-cholesterol and high-fat forage to contribute the establishment of vascular plaque. Meanwhile, safe dose of CeO_2_NPs is injected intraperitoneally to determine the prophylaxis to vascular plaque. The former and the latter is recorded as (HF, high-fat) groups and (HF + CeO_2_NPs) groups respectively.

**Fig. 6:**
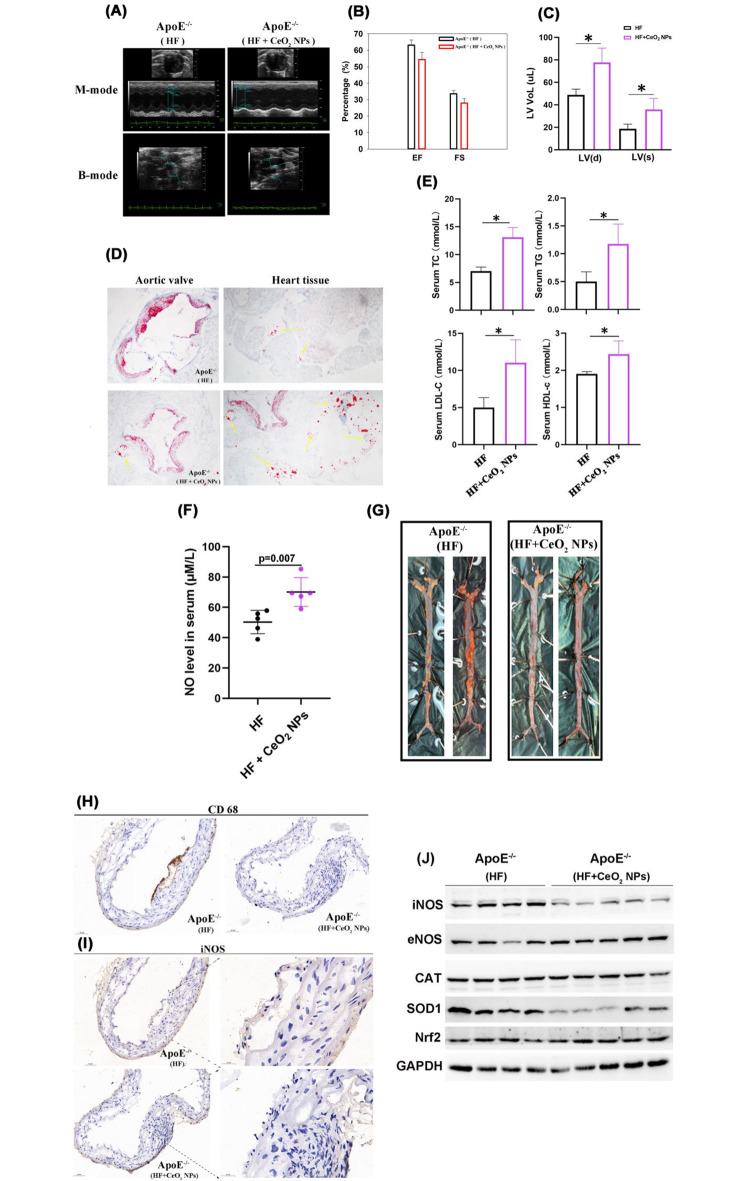
CeO_2_NPs prevent vascular plaque by NOS-like activity. (**A**) Cardiac ultrasound in living ApoE^−/−^ mice with high-fat diet. (**B**) Ejection fraction (EF), fractional shortening (FS) and (**C**) left ventricular systolic/diastolic end volume-LV(s)/LV(d) are counted according to cardiac ultrasound data. (**D**) Oil-red staining to heart aorta valve and peripheral heart tissues. (**E**) Detection of serum TC, TG, LDL-C and HDL-C in mouse. (**F**) NO concentration in ApoE^−/−^ mice serum is detected by the nitric oxide detection kit. (**G**) Mouse aortas are stripped and then stained by oil-red. (**H**-**I**) Immunohistochemical staining to the stripped aortas with CD68 antibody (**H**) and iNOS antibody (**I**). (**J**) After sacrificing the mice, arterial tissue proteins are extracted to detect the expression of iNOS, eNOS, CAT, SOD1 and Nrf2 by immunoblotting. Data is shown as mean ± SD, * *p* < 0.05, n = 5

After eight weeks of intervention with CeO_2_NPs, these mouse hearts are observed by ultrasound (Fig. 6A). Compared to HF ApoE^−/−^ mice, ejection fraction (EF) and fractional shortening (FS) in (HF + CeO_2_NPs) mice show a decline in data, but there is no significant difference (Fig. 6B). Inetrestingly, left ventricular systolic/diastolic end volume-LV(s)/LV(d) reveals a phenomenon that (HF + CeO_2_NPs) ApoE^−/−^ mice changes significantly (Fig. 6C), indicating a possibility of plaque deposition and ablation. To further demonstrate that NOS-like activity of CeO_2_NPs contributes to resist the formation of plaque in HF ApoE^−/−^ mice, the aortic valve and surrounding myocardial tissue of the mouse heart are stained with Oil-red. Results indicate that a large number of plaques are accumulated on the inner wall of blood vessel, however, the stacking density and dyeing depth are obviously assuaged after CeO_2_NPs treatment (Fig. 6D). At the same time, we observe that more free lipids is dispersed around the myocardial tissue in (HF + CeO_2_NPs) ApoE^−/−^ mice instead, but this phenomenon is not observed in HF ApoE^−/−^ mice (Fig. 6D), suggesting that CeO_2_NPs treatment ameliorates the deposition of vascular plaque by promoting blood circulation and the dispersion of blood lipids.

Additionally, we monitore the weight of mice during the experiment. CeO_2_NPs exhibit a promotion for the increased weight instead of reducing the weight of mice (Fig. S6), although it significantly improves the volume of left ventricle and the accumulation of vascular plaque stained by Oil-red. Importantly, serum TC,TG, LDL-C and HDL-C are all elevated in these treated ApoE^−/−^ mice, consistent to the higher level of serum NO level after CeO_2_NPs treatment (Fig. 6E,F). This illustrates that the improvement of vascular plaque is not achieved by improving lipid metabolism, indirectly indicating that CeO_2_NPs may promote the re-distribution of blood lipids to reduce plaque formation by its NOS-like activity. Evidences have shown that NO plays an important role in maintaining the constant tension of blood vessels, regulating the stability of blood pressure, clearing the fat and cholesterol on the blood vessel wall and affecting cell differentiation. In contrast, when similar treatment is operated on normal ICR mice, CeO_2_NPs fail to alter the trend of body weight (Fig. S7), indicating that CeO_2_NPs itself does not cause discomfort in the growth of mice. Meanwhile, blood routine data showed that the hemogram was normal after long-term treatment by CeO_2_NPs, excepting the abnormalities occurred to monocytes in two tests between intervals (Fig. S8).

In fact, these aortas stripped from (HF + CeO_2_NPs) ApoE^−/−^ mice were more negatively stained by Oil-red, comparing to the more positive staining in HF ApoE^−/−^ mice (Fig. 6G), which directly reveals a preventive effect of CeO_2_NPs on the formation of vascular plaque. These plaques are then stained with CD68 and iNOS anti-body by immunohistochemistry to detect the macrophages and the expression of iNOS in blood vessels. As shown in Fig. 6H, more CD68 positive cells are stained in these vascular plaque of HF ApoE^−/−^ mice, while there are almost no CD68 positive cells on the vascular wall of (HF + CeO_2_NPs) ApoE^−/−^ mice. Moreover, iNOS staining to vascular plaque shows a similar but weaker result, where more iNOS is expressed on vascular plaque in HF ApoE^−/−^ mice compared to that almost without expression in (HF + CeO_2_NPs) ApoE^−/−^ mice(Fig. 6I). And immunoblotting results also show a down-regulation of iNOS levels (Fig. 6J). This result is also highly consistent with the previous results observed in cell experiments, further supporting that the NOS-like activity of CeO_2_NPs triggered a unique role in preventing vascular plaque.

## Discusstion

Accordingly, we provide the evidence that CeO_2_NPs possess intrinsic NOS-like activity comparable to that of the NOS enzymatic reaction by demonstrating that (1) the reaction between CeO_2_NPs and L-Arg promote the generation of NO; (2) the NOS-like activity of CeO_2_NPs is affected by reaction time, temperature, pH, etc., this ability of CeO_2_NPs is distinctive comparing to Fe_3_O_4_NPs and AuNPs; (3) the Vo abundance of CeO_2_NPs plays an important role for its NOS-like enzymatic activity; (4) NOS-like activity of CeO_2_NPs can regulate the distribution of lipids by NO and effectively prevent the formation of vascular plaque.

Although it is unique, the NOS-like activity of CeO_2_NPs is also reasonable. Several reports have affirmed the specificity potency of CeO_2_NPs in neurodegenerative disease [33], atherosclerosis [15, 16, 31] and non- alcoholic fatty liver disease [17], etc., which is closely related to the claimed antioxidant function. However, the Vo-enriched CeO_2_ nano-rod was demonstrated as the efficient electrocatalyst with the stabilization of the crucial intermediate of *NO via inserting into vacant sites, which is conducive to the subsequent C − N coupling process rather than protonation [20]. Conversely, Janet M et al. found that CeO_2_NPs are able to scavenge NO radical and this activity of CeO_2_NPs is present in CeO_2_NPs with a lower level of Ce^3+^ state (CeO_2_NPs with a reduced number of Vo), in contrast to the superoxide scavenging properties which are correlated with an increased level of Ce^3+^ state (CeO_2_NPs with an increased number of Vo) [34]. Additionlly, Rona et al. reported CeO_2_NPs can induce the generation of NO from S-nitrosoglutathione and maintain a high NO release recovery rate. These evidences more or less corroborated the fact that CeO_2_NPs have the ability to regulate or participate in the regulation of NO generation or conversion, as well as the potential in imitating NOS to catalyze L-Arg [21].

Further, given the pivotal role of Vo in CeO_2_NPs for exerting its NOS-mimicking activity, it is intriguing to inquire whether or not a superior catalytic efficacy can be realized via the manipulation of other elements in the synthesis of CeO_2_NPs. Evidences have revealed that incorporation of zirconium into CeO_2_NPs results in an elevated proportion of Ce^3+^, with the expectation being that these more Vo in a crystal lattice will contribute to enhancing its NOS-mimicking capacity [13, 35]. While the generated NO by NOS-like activity of CeO_2_NPs undoubtedly aids in averting vascular plaque and atherosclerosis, unrestricted generation of NO carries inherent dangers for our bodies [36, 37]. As revealed in Fig. S8, CeO_2_NPs induced an abnormal elevation in monocytes in normal mice. This highlights the potential biocompatibility concerns of nanoparticles, which may, to a certain extent, provoke inflammatory reactions, although a large number of studies have shown that CeO_2_NPs has good biosafety [38, 39]. Additonally, the monitoring of blood pressure in mice also suggests another possibility (data not shown), although the blood pressure statistics did not show significant differences between groups, the slight decrease in average blood pressure may be attributed to the vasodilation effect of CeO_2_NPs derived NO on vascular smooth muscle. However, in this study, we cannot rule out the potential biosafety issues that may cause such changes. Before any potential progression to clinical applications, extensive research remains to be conducted, focusing areas such as the influence of nanoparticles’ dimensions, surface modifications, colloidal stability and possible cytotoxicity, etc.

Generally, CeO_2_NPs is recognized as a candidate for multi-disease based on its excellent redox regulation [40]. However, in relieving atherosclerosis and targeting to vascular plaque, the declared anti-ROS function seems to be magnified, although this is also one of the extremely important factors [5]. Vascular plaques are closely related to blood lipids, when blood lipids are abnormal, the concentration of low density lipoprotein cholesterol (LDL-C) will increase, more LDL-C wil drive inflammatory response [41–43]. Macrophages that swallow too much cholesterol will transform into foam cells and deposit in the intima of blood vessels, forming a yellowish substance [44]. Thus, abnormal blood lipid and foam cell deposition are the more critical causes for the formation of vascular plaque [44]. These pathogenic factors will feed back to inhibit NO secretion in endothelial cells, leading to a vicious circle [45, 46]. Because in addition to vasodilation and improving blood flow, NO can also take away fat and cholesterol accumulated on the blood vessel wall [42, 43].

Therefore, we observed that CeO_2_NPs can significantly reduce the formation of plaque on the vascular wall, which may be beyond the reach of simple anti-ROS, although we did not shield its anti-ROS process, in fact, it is also difficult to completely shield it. Anyway, we prefer to believe that the outstanding potential of CeO_2_NPs is the result by joint action of anti-ROS and other mechanisms, which includes its important but covert NOS-like enzymatic activity.

## Conclusion

CeO_2_NPs substantially exhibit nitric oxide synthase (NOS) functionality via catalyzing the transformation of L-Arg into nitric oxide or the derivatives. This NOS-mimicking potency of CeO_2_NPs is ascribed to an array of variables, particularly oxygen vacancies. The NOS-like property of CeO_2_NPs contributes to augmenting the release of nitric oxide from vascular endothelial cells and macrophages without depending on NOS level. And the NOS-simulating ability of CeO_2_NPs is conducive in escalating endogenous nitric oxide levels, lipid repositioning and consequently diminishing vascular plaques.

### Electronic supplementary material

Below is the link to the electronic supplementary material.

Supplementary Material 1

## Data Availability

The data that support the findings of this study are available from the corresponding author upon reasonable request.
