# Supplementary material for: NOS-like activity of CeO2 nanozymes contributes to diminishing the vascular plaques
Source: J Nanobiotechnology. 2024 Jan 3;22:12. doi: 10.1186/s12951-023-02276-5 (PMC10763164; doi:10.1186/s12951-023-02276-5)
Supplement: Supplementary file 1 — Supplementary Material 1 [file 12951_2023_2276_MOESM1_ESM.docx]

**NOS-like Activity of CeO_2_ Nanozymes Contributes to Diminishing the Vascular Plaques**

Yuxiang Sun^1^*, Tianze Xu^2^, Yike Qian^1^, Qiaoyun Chen^1^, Fei Xiong^3^,

Wenxian Du^4^*, Li Xu^1^*

1. Institute of Translational Medicine, Medical College, Yangzhou University, Yangzhou, 225001, PR China; Jiangsu Key Laboratory of Integrated Traditional Chinese and Western Medicine for Prevention and Treatment of Senile Diseases, Yangzhou University, Yangzhou, PR China.

2. Department of Vascular Surgery, the Affiliated Drum Tower Hospital of Nanjing University Medical School, China

3. State Key Laboratory of Bioelectronics, Jiangsu Key Laboratory for Biomaterials and Devices, School of Biological Science and Medical Engineering & Collaborative Innovation Center of Suzhou Nano-Science and Technology, Southeast University, Nanjing 210096, People’s Republic of China.

4. Institute of Diagnostic and Interventional Radiology, Shanghai Sixth People's Hospital, School of Medicine, Shanghai Jiaotong University, No. 600, Yishan Road, Xuhui District, Shanghai 200233, China

* Corresponding author:

Yuxiang Sun, [sunyuxiang@yzu.edu.cn](mailto:sunyuxiang@yzu.edu.cn)

Li Xu, xulibg@yzu.edu.cn

Wenxian Du, wx0910@mail.ustc.edu.cn

**SUPPORTING FIGURES**


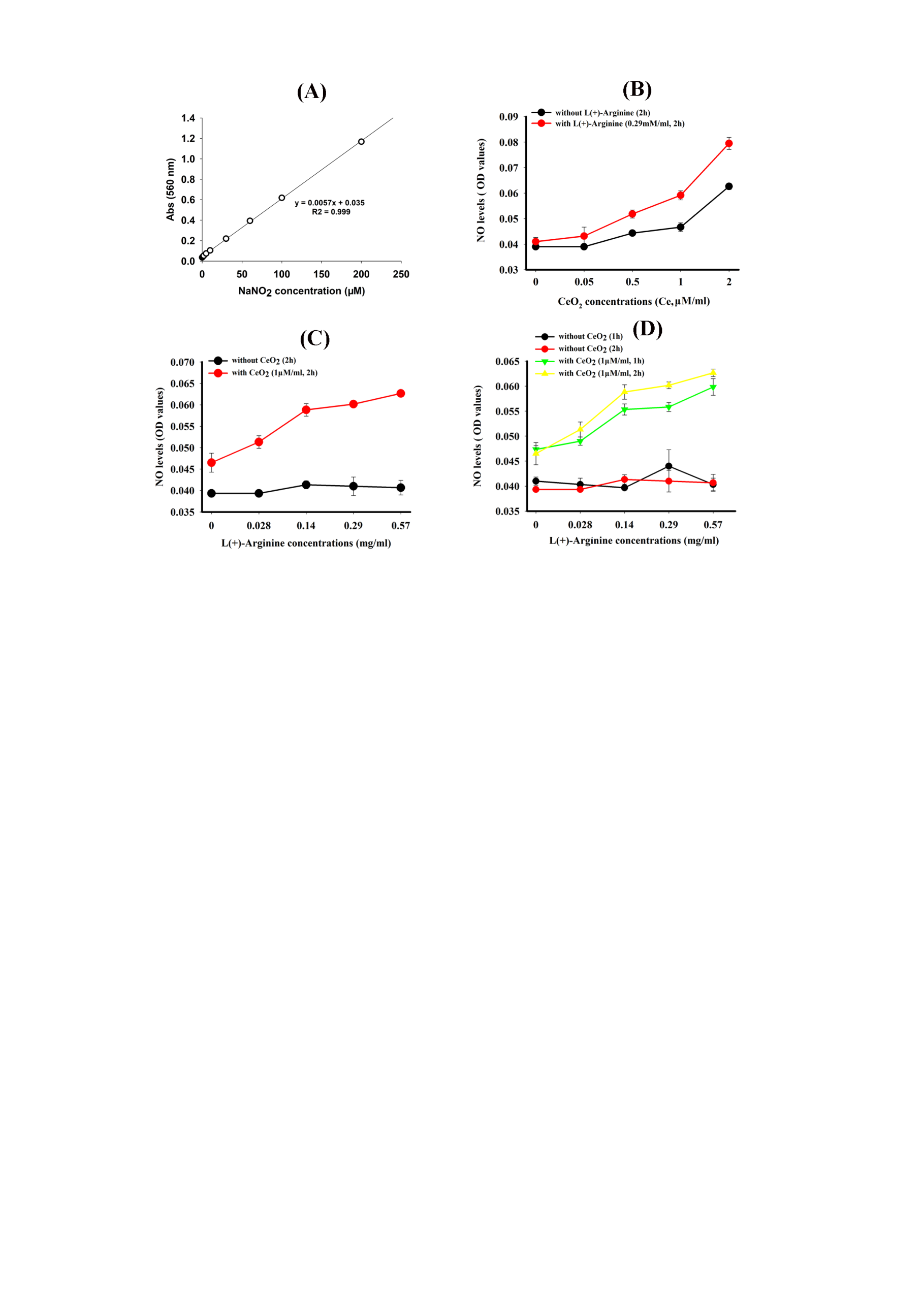


**Fig.S1** (A) Standard curve of NO detection is based on Griess Reagents by counting the absorbance at 560nm. (B) The concentration of L-Arg was fixed at 0.29 mg/mL, and then NO level was measured in CeO_2_NPs/L-Arg system with different CeO_2_NPs concentrations. (C) The concentration of CeO_2_NPs is fixed at 1 mM, and NO level was measured in CeO_2_NPs/L-Arg system with different L-Arg concentrations. (D) According to the conditions in (C), NO detection was carried out after reaction for 1h and 2h respectively.


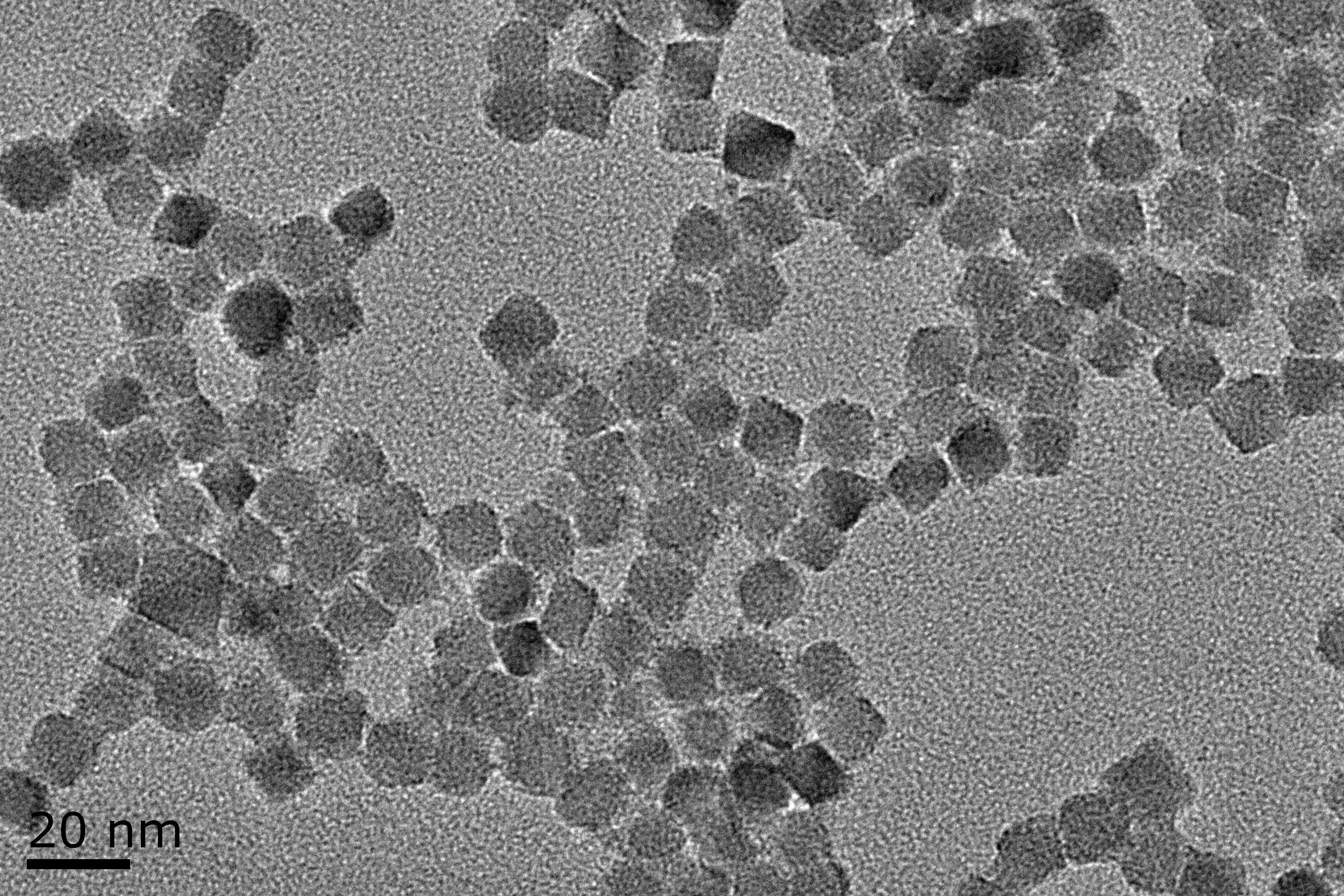


**Fig.S2** TEM of Fe_3_O_4_@CA NPs.


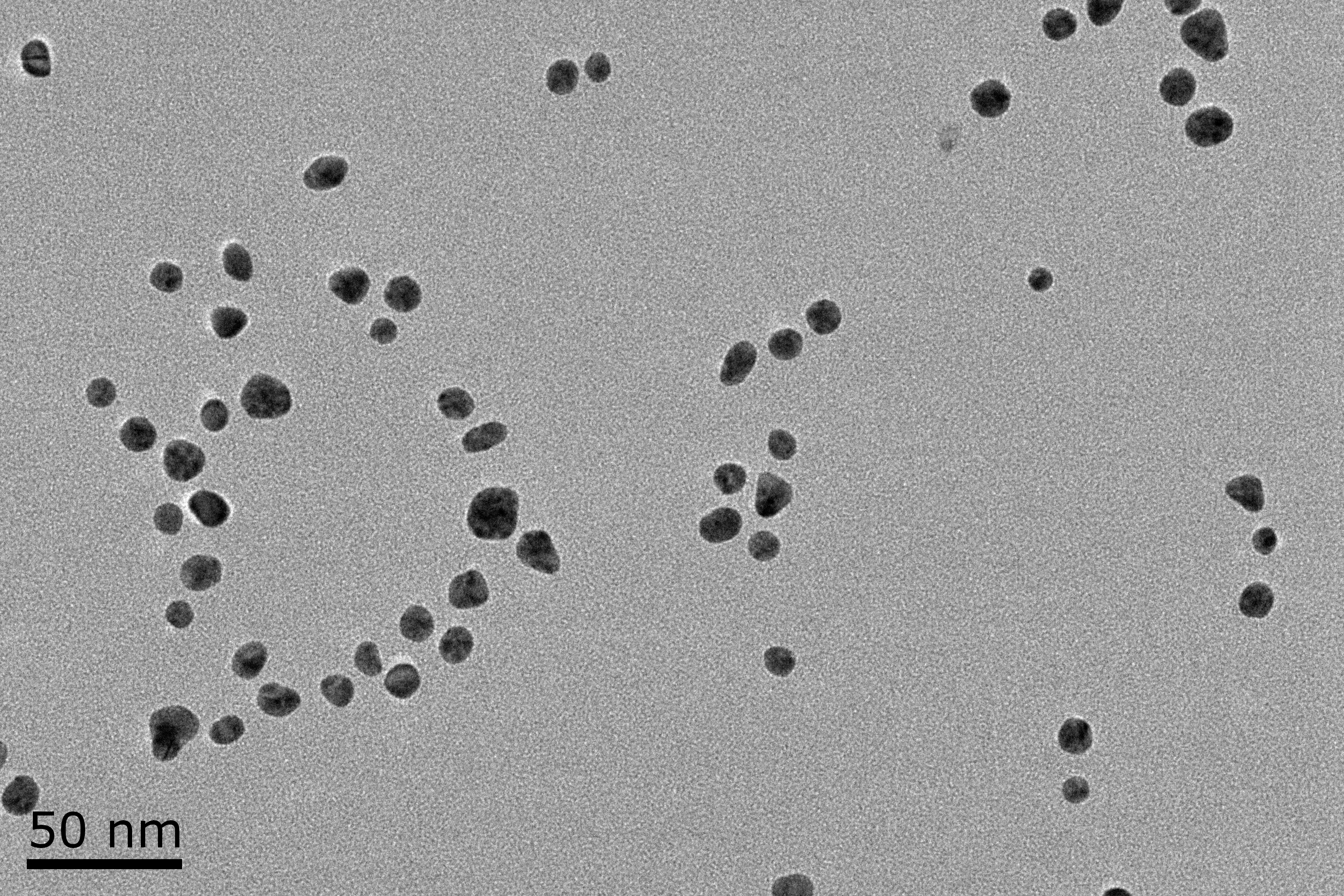


**Fig.S3** TEM of Au@Dextran NP_S_.


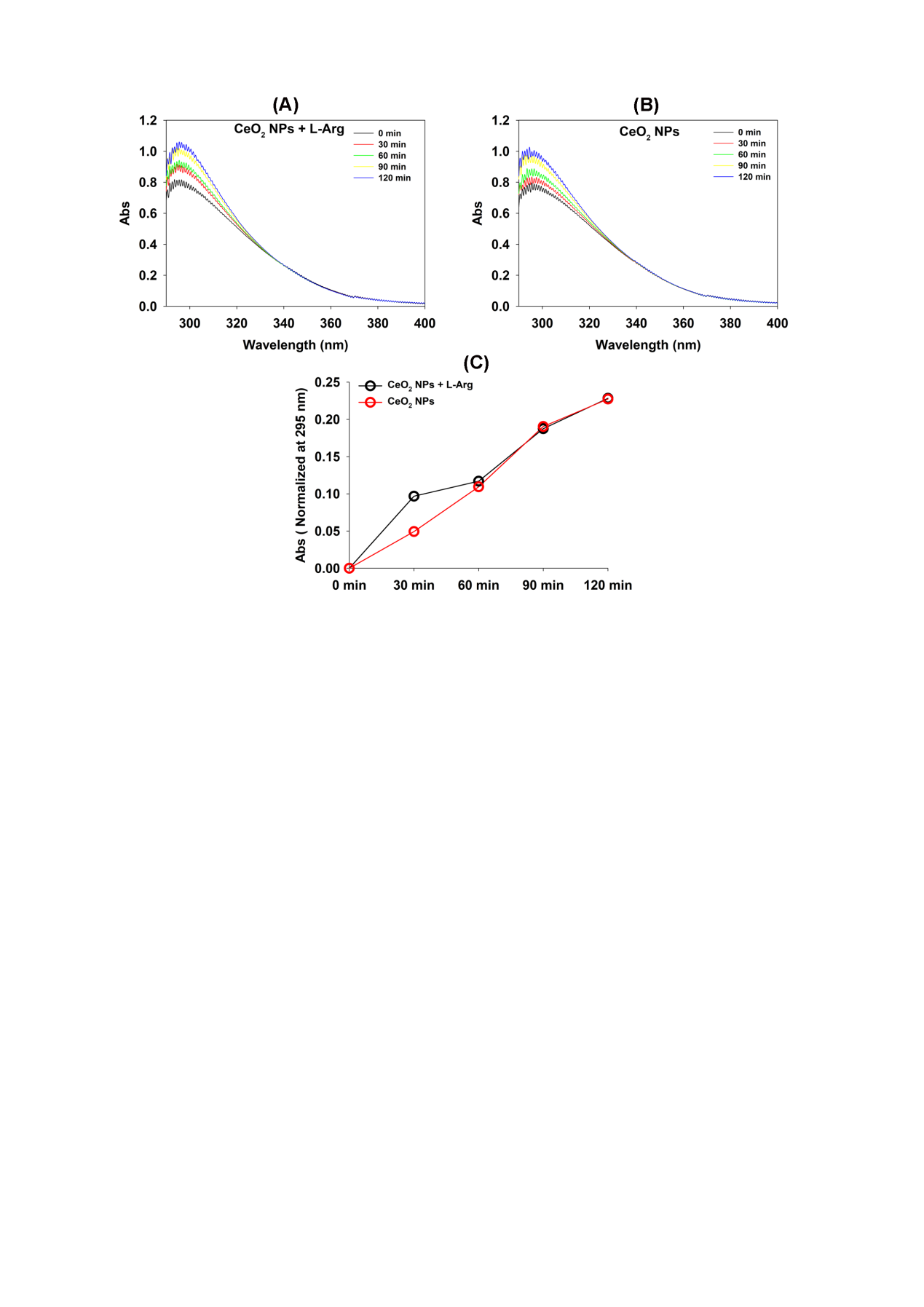


**Fig.S4** UV-Vis spectra of CeO_2_NPs plus L-Arg (A) and CeO_2_NPs (B) in PBS buffer with time. (C) Calculating the absorbance at 295 nm in response to Ce^4+^ shift.


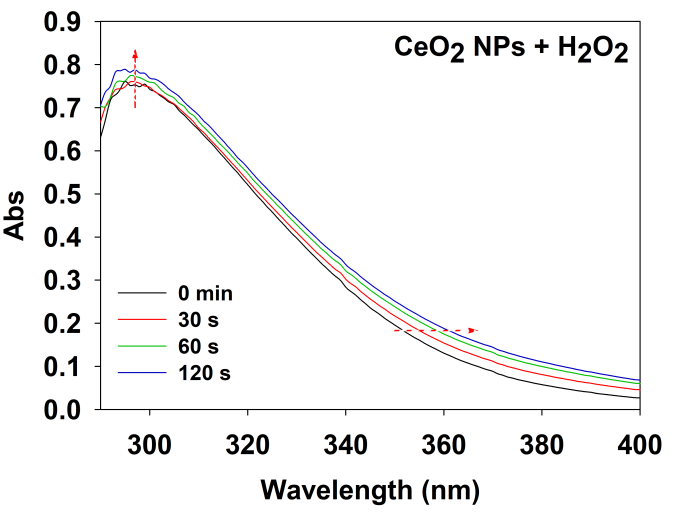


**Fig.S5** UV-Vis spectra of CeO_2_NPs (1mM) plus H_2_O_2_ (50 μL, 3%H_2_O_2_) in PBS buffer (2mL) with time, the curve responses to the increase at 295 nm absorbance and the red shift.


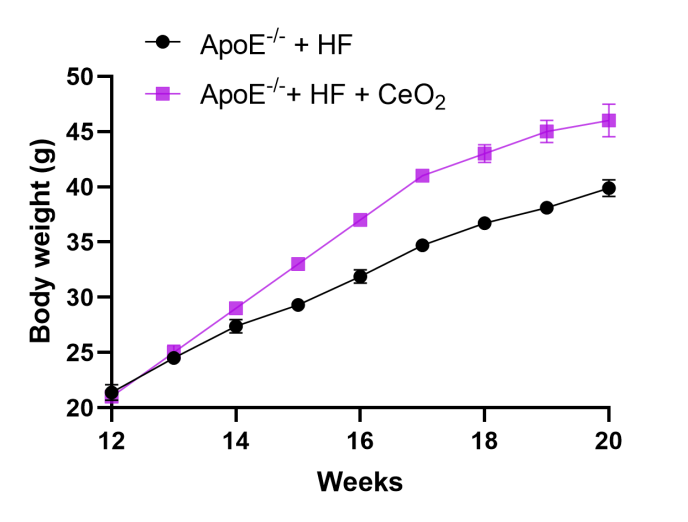


**Fig.S6** Body weight of ApoE^-/-^ mice feed with high-fat frage for 8 weeks and treated by CeO_2_NPs.


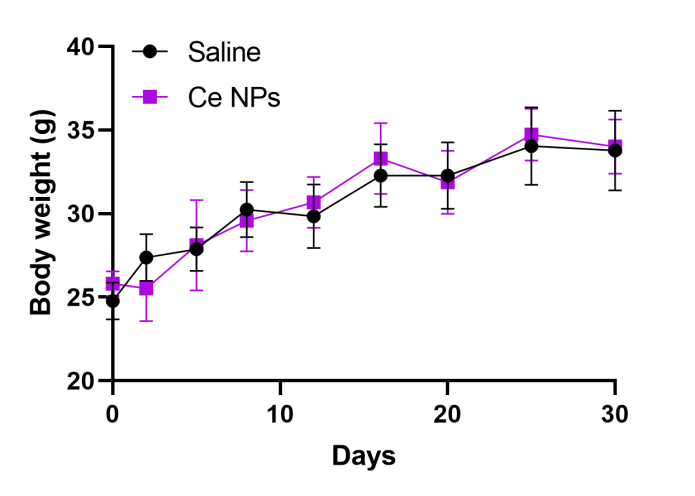


**Fig.S7** Body weight of ICR mice on a normal diet for 30 days and treated by CeO_2_NPs.


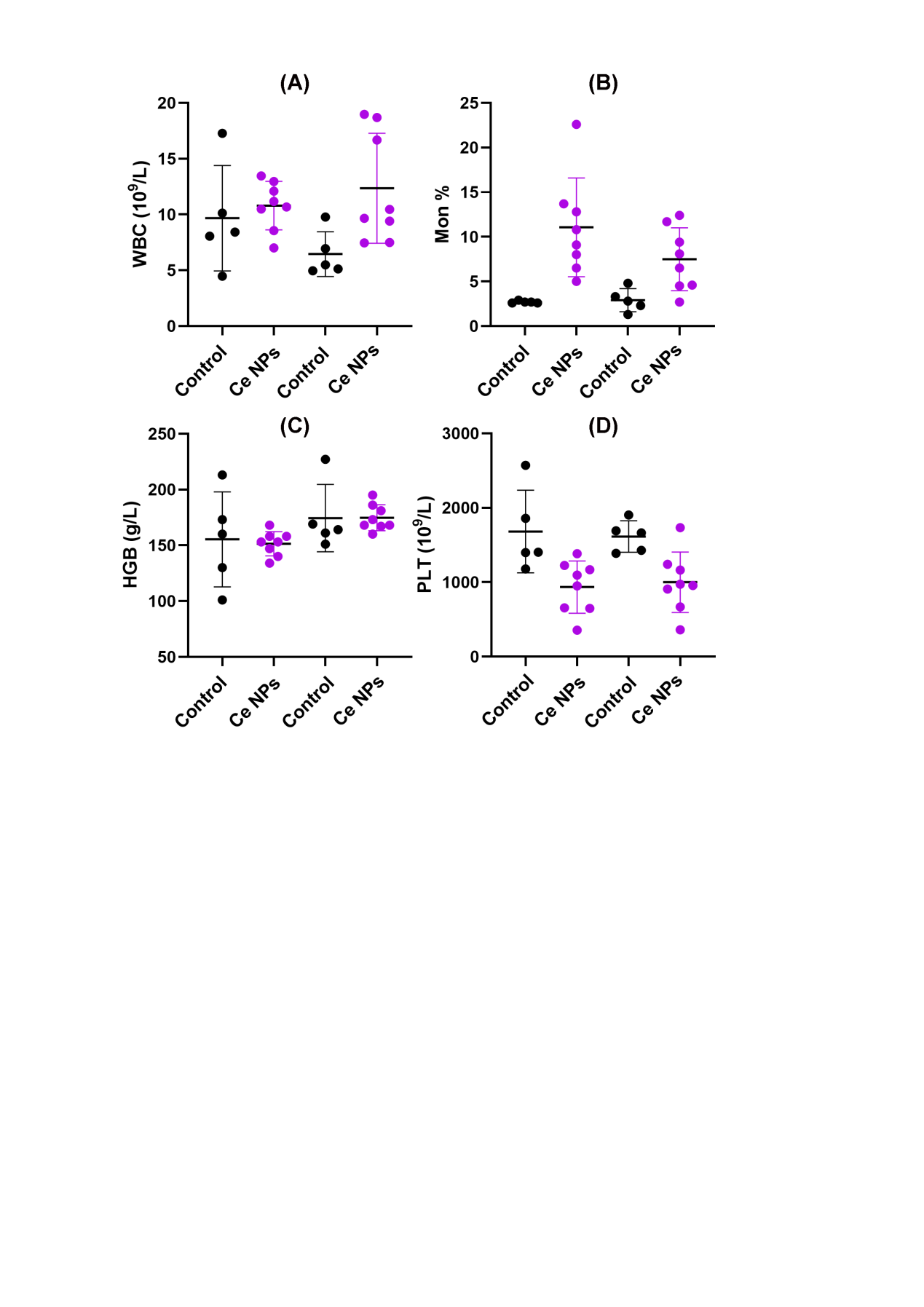


**Fig.S8** ICR mouse blood routine data that was measured on the 14th and 30th days after CeO_2_NPs treatment, respectively
